# Auxiliary medial small locking plate fixation enhances bone healing and minimizes delayed union in inverted V‐shaped high tibial osteotomy: A comparative study of 154 knees

**DOI:** 10.1002/jeo2.70919

**Published:** 2026-09-23

**Authors:** Takuma Kaibara, Kazunori Yasuda, Eiji Kondo, Koji Yabuuchi, Masayuki Inoue, Norimasa Iwasaki, Tomonori Yagi

**Affiliations:** ^1^ Department of Orthopaedic Surgery Yagi Orthopaedic Hospital Sapporo Japan; ^2^ Department of Orthopaedic Surgery Faculty of Medicine and Graduate School of Medicine, Hokkaido University Sapporo Japan; ^3^ Centre for Sports Medicine Hokkaido University Hospital Sapporo Japan

**Keywords:** bone healing, delayed union, high tibial osteotomy, inversed V‐shaped osteotomy, locking plate fixation

## Abstract

**Purpose:**

To determine whether auxiliary fixation with a T‐shaped small locking plate (SLP) placed on the medial tibial cortex can enhance bone healing and reduce delayed union compared with the conventional inverted V‐shaped high tibial osteotomy (iVHTO) fixed with a locking compression plate (LCP).

**Methods:**

This retrospective cohort study included 154 knees (135 patients) that underwent iVHTO. The knees were allocated to Group S (*n* = 72), treated with conventional single LCP fixation, and Group D (*n* = 82), treated with auxiliary medial SLP fixation in addition to the conventional procedure. Radiological and clinical evaluations were performed with a minimum follow‐up of 2 years. Bone healing was assessed using radiographs and computed tomography scans, and the time to complete union was quantitatively evaluated with an established scoring system.

**Results:**

The time to union was significantly shorter in Group D than in Group S (median, 8 vs. 12 weeks; *p* < 0.001; 95% confidence interval [CI] for the difference, 0.0–4.0 weeks). The incidences of delayed union and non‐union were both significantly lower in Group D than in Group S (2.4% vs. 20.8% and 0% vs. 5.6%, respectively; *p* < 0.001 and *p* = 0.046; 95% CIs, 8.6%–29.3% and 0.0%–13.4%). The rate of other complications, including transient anterior compartment syndrome and superficial infection, did not differ significantly between the groups (4.9% vs. 4.2%; *p* = 0.98; 95% CI, –7.2% to 8.2%). The Lysholm score was slightly but significantly higher in Group D than in Group S (*p* = 0.043; 95% CI, 0.0–5.0), whereas no significant differences were observed in the other clinical scores.

**Conclusion:**

Auxiliary medial fixation using an SLP enhanced bone healing and minimized the incidence of delayed union or non‐union following conventional iVHTO with single lateral LCP fixation, without increasing other complications or compromising clinical outcomes.

**Level of Evidence:**

Level III, prospective cohort study.

**Abbreviations**
aORs: adjusted odds ratios
AP: anteroposterior
BMD: bone mineral density
BMI: body mass index
CI: confidence interval
CT: computed tomography
FTA: femorotibial angle
HKA: hip–knee–ankle
HTO: high tibial osteotomy
IQR: interquartile range
iV: inverted V‐shaped
JOA: Japanese Orthopaedic Association
KOOS: Knee Injury and Osteoarthritis Outcome Score
LCP: locking compression plate
LCW: lateral closing wedge
MA: mechanical axis
MOW: medial opening wedge
MPTA: medial proximal tibial angle
MRI: magnetic resonance imaging
OA: osteoarthritis
OAT: osteochondral autograft transfer
PTS: posterior tibial slope
SD: standard deviation
SLP: small locking plate
YAM: young adult mean

## INTRODUCTION

High tibial osteotomy (HTO) is a useful surgical option for active patients with a medial osteoarthritis (OA) of the knee who wish to continue participating in sports or performing strenuous labour. Medial opening wedge (MOW) HTO is a well‐established procedure for knees with mild OA and mild varus deformity [3, 10]. However, MOWHTO has been reported to carry a higher risk of complications, such as lateral hinge fracture, non‐union, residual varus deformity and patella baja, in patients with varus deformity requiring a large correction [7, 8, 9, 22, 27, 41]. Therefore, for such patients, lateral closing wedge (LCW) HTO has often been selected. Although long‐term outcomes of LCWHTO have been reported to be favourable [29, 36, 38, 44], this procedure carries a higher risk of complications different from MOWHTO, such as peroneal nerve palsy, non‐union, bone loss from the proximal tibia, patellar height changes and leg‐length discrepancy [1, 25, 35, 36]. In addition, LCWHTO has been reported to carry a higher risk of delayed union [1, 37, 38], although the details of delayed union have rarely been investigated in previous studies. The inverted V‐shaped (iV) HTO procedure, defined as a combination of hemi‐LCW and hemi‐MOW HTO with immediate bone grafting, was developed to reduce the drawbacks of LCWHTO [1, 19]. In the original inverted V‐shaped high tibial osteotomy (iVHTO) procedure, an external fixator was used [1]. Kondo et al. [19] reported a modified iVHTO procedure, in which the osteotomy site was fixed with a laterally placed locking compression plate (LCP). The iVHTO is classified as a neutral‐wedge HTO because the centre of tibial alignment correction in this procedure is located approximately at the centre of rotation of angulation (CORA) of the tibia [21, 31]. Therefore, the iVHTO procedure with LCP fixation minimizes changes in patellar height, posterior tibial slope (PTS), tibial length and the bone mass and morphology of the proximal tibia [18]. However, few studies have investigated the time required to achieve complete bone healing following the iVHTO procedure.

Recently, Yabuuchi et al. [42] reported that the incidence of delayed bone union at the osteotomy site after this procedure was approximately 10%, a rate considered clinically unsatisfactory. In response to this issue, the authors developed a minimally invasive technique in which a T‐shaped small locking plate (SLP) is placed on the medial tibial cortex using a percutaneous approach during iVHTO. The purpose of this study was to determine whether auxiliary fixation with an SLP on the medial tibial cortex can enhance bone healing and reduce the incidence of delayed union compared with the conventional iVHTO procedure. The authors hypothesized that (1) auxiliary fixation with a SLP on the medial tibial cortex would shorten the time to bone union and reduce the incidence of delayed union and non‐union compared with the conventional iVHTO procedure using a single LCP fixation; and (2) this additional fixation would not increase the rates of other complications or compromise clinical outcomes of the conventional iVHTO procedure.

## METHODS

### Study design

The retrospective cohort study protocol was approved by the institutional ethics review board (No. H29‐0001). All patients were informed about the use of their clinical data regarding surgical treatment for clinical research purposes, and written informed consent was obtained from all patients.

The indications for the iVHTO procedure were as follows: (1) A medial compartment (tibiofemoral) OA knee (Kellgren–Lawrence [16] Grade 1–4) with persistent knee pain refractory to at least 3 months of conservative treatment. Grade‑1 knees indicated for this procedure included cases with marked varus alignment in which both MMPRT and osteonecrosis were confirmed on magnetic resonance imaging. (2) A patient's desire to return to high‐demand daily activities, sports or heavy labour. In addition, regarding the correction angle during HTO, the indications for the iVHTO procedure included either (3) a medial OA knee without patellofemoral OA that requires a valgus correction exceeding 10° to achieve the target alignment (%MA, 66.7%) or (4) a medial OA knee with moderate (Grades 2–3) patellofemoral OA, regardless of the planned valgus correction angle. The contraindications for the iVHTO procedure were as follows: (1) flexion contracture exceeding 10°, (2) maximum knee flexion less than 120°, (3) meniscal and/or chondral lesions in the lateral compartment, (4) severe (Grade 4) patellofemoral OA, (5) cruciate or collateral ligament insufficiency, (6) history of deep infection in the knee or tibia and (7) history of severe trauma to the lower limb.

The inclusion criteria for this study were knees that underwent iVHTO at our institution between January 2018 and October 2023, according to the above‐described indications and contraindications. However, patients aged 72 years or older were excluded from this study. The postoperative exclusion criteria included (1) patients who developed functional impairment in the lower limb other than the operated knee during the follow‐up period and (2) patients who experienced incidental medical conditions or trauma after surgery.

**Figure 1:**
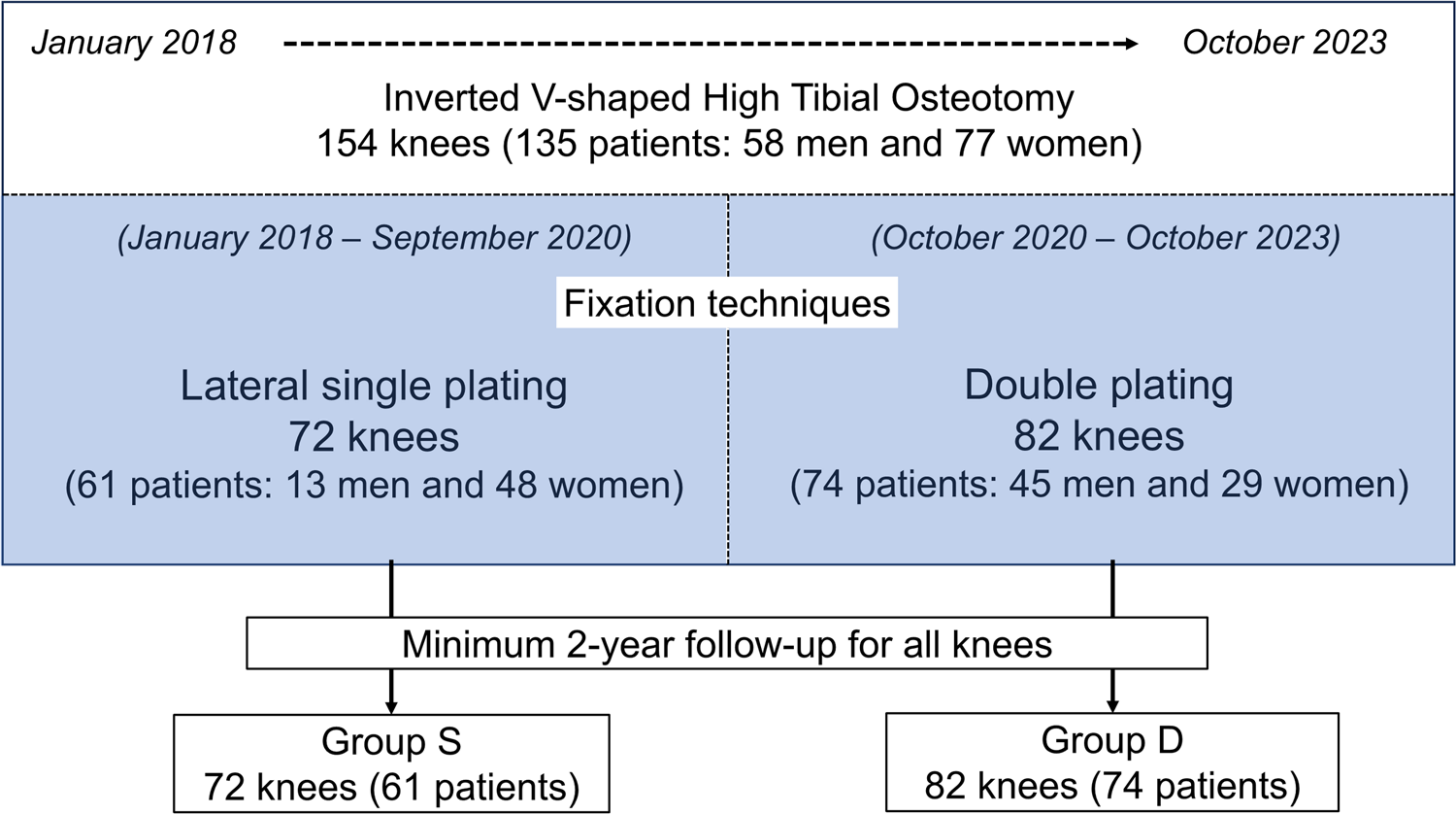
Flowchart of the patients in the two study groups. All 154 knees of 135 patients could be followed up for a minimum of 2 years in our outpatient clinic.

**Figure 2:**
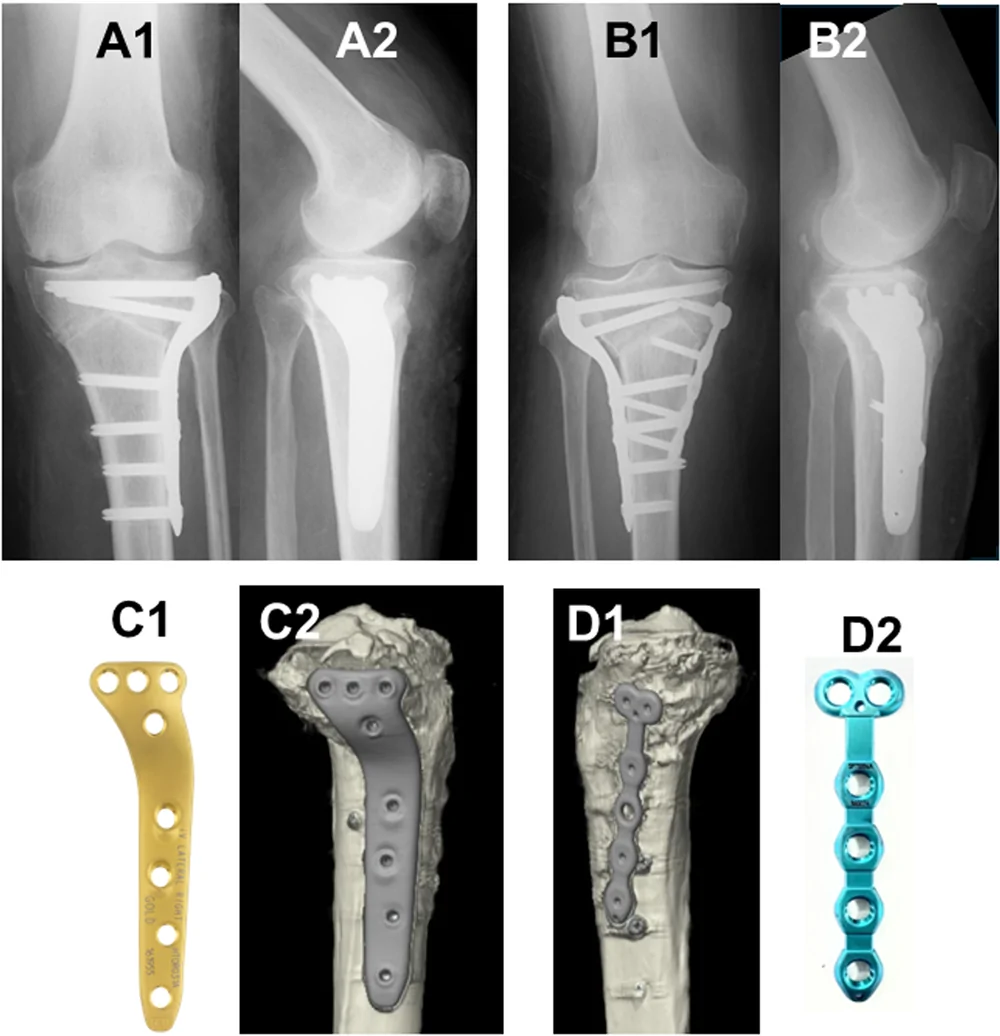
Postoperative radiographs of conventional single‐plating technique (a1, a2) and double‐plating technique (b1, b2). (c1, c2) Locking compression plate fixed with 5.5‐mm cannulated locking screws. (d1, d2) Small locking plate fixed with 4.2‐mm cannulated locking screws. (b1, b2, c2, d1) Images obtained from the same knee.

A total of 154 knees in 135 patients met the eligibility criteria and were enroled in this study. The study period was chronologically divided into two phases (Figure 1). Between January 2018 and September 2020, 72 knees in 61 patients (Group S) underwent iVHTO using the conventional single plating technique (Figure 2a1,a2,c1,c2). In this technique, the osteotomy site was fixed with a laterally placed LCP (TriS Inverted‐V Plate System; Osferion Biomaterials). Based on our clinical assessment, we subsequently determined that the incidence of delayed union after this surgery was not clinically acceptable. Therefore, we began applying the double plating technique in October 2020 to reduce this incidence (Figure 2b1,b2). In this technique, medial auxiliary fixation with an SLP (TriS small plate system; Osferion Biomaterials) was added to the conventional lateral fixation (Figure 2d1,d2). Consequently, 82 knees in 74 patients (Group D) underwent iVHTO using the double plating technique by October 2023. All operations were performed by two senior orthopaedic surgeons (K.Y. and E.K.) experienced in these procedures. An identical postoperative rehabilitation protocol was used for all patients. All patients were followed in the outpatient clinic for a minimum of 24 months (Figure 1). For patients who had a follow up period exceeding 36 months, the data evaluated at 24–36 months postoperatively were used for comparative analysis to ensure that the observation periods were aligned between the two groups. Accordingly, the follow‐up data for all patients were obtained at 24–36 months after surgery. The two groups were compared in terms of patient demographics and baseline clinical characteristics, radiographic assessment and clinical evaluation.

### Surgical procedures

#### Preoperative planning

The tibial valgus correction angle is determined using the Miniaci's method [28] based on a full‐length standing anteroposterior (AP) radiograph. A lateral wedge resection angle was determined for each knee so that the mechanical axis in the corrected limb passed through a point corresponding to 66.7% of the tibial plateau width measured from the medial edge.

#### iVHTO procedure fixed with a lateral LCP

The details of the iVHTO procedure have been previously reported [14, 19]. First, fibular osteotomy was performed at the centre of the shaft [39, 43]. Under fluoroscopic guidance, a K‐wire was inserted at the preoperatively planned apex point of the V‐shaped osteotomy, perpendicular to the anterior surface of the proximal tibia. A wire insertion guide, set at the preoperatively planned correction angle, was attached to the apex K wire, and a pair of guide K wires was inserted toward the apex wire through this guide. After a coronal ascending osteotomy was performed, a lateral hemi‐wedge osteotomy was then made along the two K wires. A parallel drill guide was mounted on the apex K wire, and multiple 2 mm parallel holes were created in the medial tibia. A medial hemi‐osteotomy was performed along these drill holes. After valgus correction of the tibia, the osteotomy site was fixed with an LCP placed on the lateral side [14]. Finally, morselized bone chips prepared from the resected wedge were implanted into the medial opening gap. Finally, the fibular osteotomy site was reduced and ligated with a suture [39, 43].

#### Medial auxiliary fixation technique

**Figure 3:**
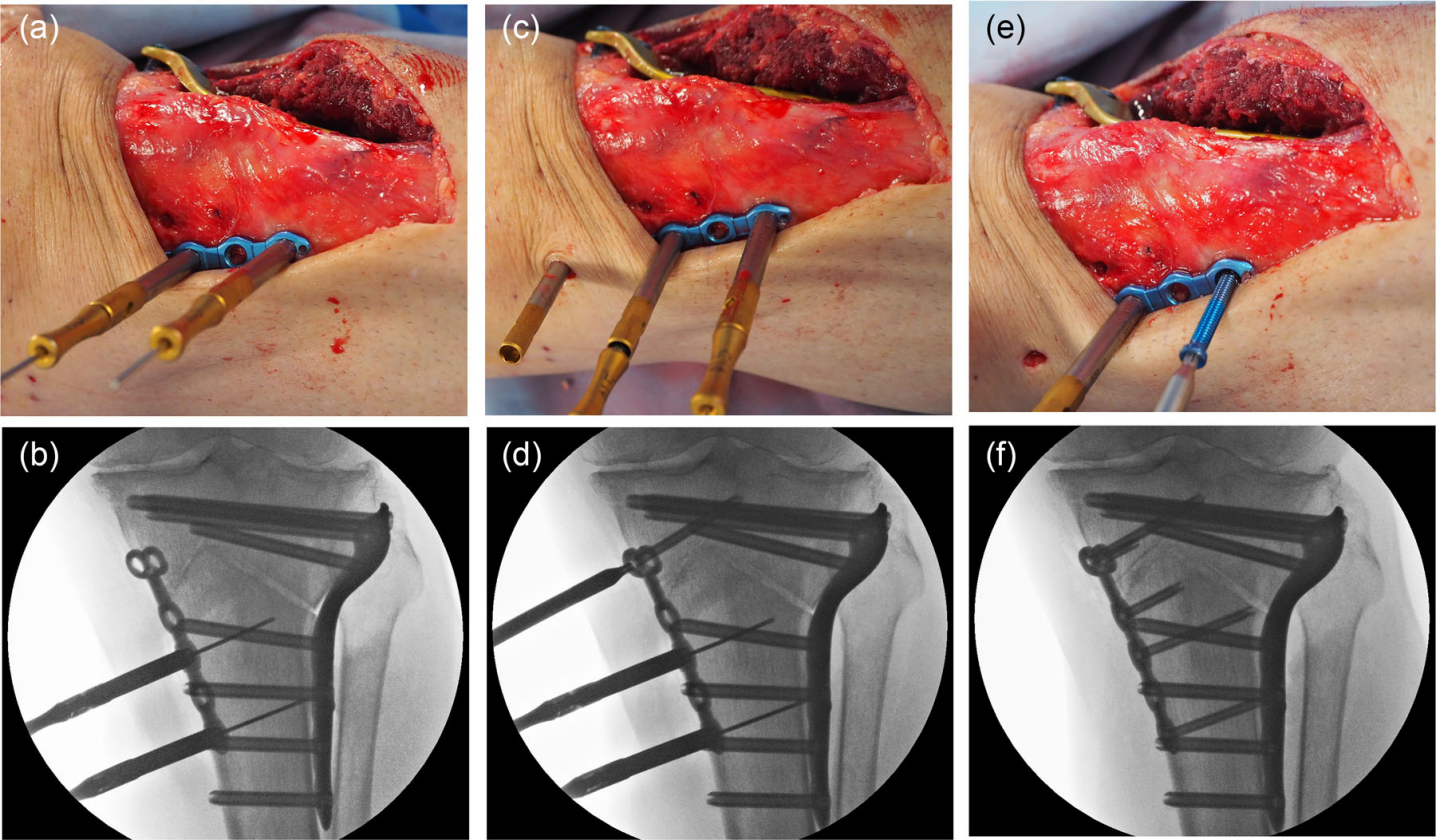
Medial auxiliary fixation technique with a small locking plate (SLP). (a, b) An SLP was placed on the tibia and secured temporarily with two K‐wires. (c) A drill sleeve was attached to a proximal hole through a small incision. (d) A cannulated locking screw was inserted into the tibia through the small incision. (e, f) The distal part of the SLP was secured with locking screws.

The SLP was placed on the anteromedial aspect of the proximal tibia through the existing lateral incision. Under fluoroscopic guidance, the position of the SLP was decided so that the screws to be inserted for fixation would not interfere with the screws already placed in the lateral plate. The SLP was temporarily fixed with two wires inserted into the tibia through the pre‐attached sleeves (Figure 3a,b). A 1‐cm skin incision was made over the proximal part of the SLP. Through this incision, a drill sleeve was attached to one of the SLP holes (Figure 3c), and the tibia was subsequently drilled through the sleeve. A guide wire was inserted into the hole, and a 4.2‐mm‐diameter cannulated locking screw was then inserted into the tibia over the guide wire (Figure 3d). This technique was repeated to insert the remaining proximal screws. Finally, the distal part of the SLP was secured with two or three locking screws (Figure 3e,f).

### Postoperative management

Postoperative management in both groups followed the same previously reported protocol [18, 19]. Quadriceps setting and straight leg raising exercises were initiated on postoperative Day 1. Passive range of motion (ROM) was restricted to 0°–90° during the first 2 weeks. Active ROM was progressed to 0°–120° from 3 weeks, and full ROM was permitted from 6 weeks. Partial weight‐bearing with crutches was permitted at 3 weeks, and full weight‐bearing was authorized at 5 weeks.

### Patient demographics and baseline clinical characteristics

Baseline patient characteristics included sex, age, height, body weight, body mass index (BMI), bone mineral density (BMD), OA grade and concomitant surgical procedures. BMD was measured using a DXA bone densitometer (Discovery A; Hologic Inc.) and expressed as a percentage of the young adult mean (YAM). These baseline characteristics were compared between Groups S and D.

### Radiological assessments

#### Alignment of the lower limb

Standard AP and lateral knee radiographs were obtained preoperatively and at 1, 5, 8, 12, 16, 20, 24 and 36 weeks postoperatively to determine the timing of bone union and to detect delayed union at an early stage, based on the findings of the preceding study [42]. In addition, radiographs were taken at 52 and 104 weeks as part of routine follow‐up examinations. Computed tomography (CT) scans were obtained at 5, 12 and 24 weeks; however, to minimize radiation exposure, further CT assessments were discontinued once bone union had been confirmed [42]. OA severity was graded according to the Kellgren–Lawrence classification [16]. Lower limb alignment and tibial morphology were evaluated on AP radiographs by measuring the anatomical femorotibial angle (FTA), hip–knee–ankle (HKA) angle, percentage of the mechanical axis (%MA) and anatomical medial‐proximal tibial angle (MPTA). On lateral radiographs, the PTS and Insall‐Salvati ratio [13] were measured. These parameters were measured using dedicated planning software (MediCAD; Hectec).

#### Bone healing and time to union assessment

Progression of bone healing at the lateral osteotomy site after iVHTO was radiologically classified into three types (direct healing, indirect healing and osteolysis) according to Yabuuchi's criteria [42] (Figure S1). The detailed criteria for each type, defined using radiographs and CT images, are summarized in Table S1. Time to bone union at the osteotomy site was quantitatively assessed using the modified RUST (Radiographic Union Score for Tibial fractures) system [40, 42] (Table S2). The time to bone union was defined as the point at which either of the two scoring systems reached a full score of 12 points. Delayed union was defined as the absence of completed bone union at the osteotomy site 16 weeks after surgery [42]. Non‐union was defined as persistent lack of bone union at 36 weeks [23, 34]. In addition, knees requiring additional surgery to treat a large radiolucent lesion were classified as non‐union irrespective of postoperative duration.

Two experienced orthopaedic surgeons (T.K. and K.Y.), who were blinded to clinical symptoms at each follow‐up, independently evaluated all images. When their assessments of the discrete scores differed, they reviewed the CT findings together and reached a consensus on the final score. Inter‐rater reliability was calculated between the two raters. Intra‐rater reliability was determined from two assessments performed by the second rater, with an 8‐week interval between them.

#### Clinical evaluations

During surgery, the additional operative time required for SLP placement in Group D was recorded using a stopwatch by operating‐room nurses who were independent of the study. Peri‐ and postoperative complications were recorded in the electronic medical records. Clinical symptoms and knee function were evaluated preoperatively and at a minimum of 2 years postoperatively using the Japanese Orthopaedic Association (JOA) score [30], the Lysholm score [24] and the Knee Injury and Osteoarthritis Outcome Score (KOOS) [33].

### Statistical analysis

Inter‐ and intra‐rater reliability for the radiographic union score was assessed using Cohen's *κ* and quadratic‐weighted kappa statistics. Continuous variables were assessed for normality using the Shapiro–Wilk test. Normally distributed data were expressed as the mean ± standard deviation (SD), while non‐normally distributed data were expressed as the median [interquartile range (IQR)]. Differences between groups were analysed using the Student's *t* test for normally distributed continuous variables and the Mann–Whitney *U* test for non‐normally distributed variables. Categorical variables were compared using the Pearson *χ* ^2^ test or Fisher's exact test, as appropriate. The 95% confidence interval (CI) for the difference in the incidence of delayed union between the two groups was calculated using the Newcombe–Wilson method. The odds ratio and its 95% CI were also calculated. To evaluate the therapeutic effects of HTO, preoperative and postoperative data were compared using the paired *t* test or the Wilcoxon signed‐rank test.

To identify independent factors associated with postoperative delayed bone healing, a multivariate logistic regression analysis was performed. The model included the following variables: surgical technique, sex, BMI, BMD and the intraoperative correction angle. These covariates were selected to determine whether the clinical parameters that showed significant differences between the two groups were independent factors associated with postoperative delayed union, based on their clinical relevance to bone healing [2, 15]. The results were expressed as adjusted odds ratios (aORs) with 95% CIs. Given the limited number of events, the model was restricted to five clinically relevant covariates that were selected a priori based on previous literature on risk factors for delayed bone union after HTO [2, 15].

All statistical analyses were performed using IBM SPSS Statistics version 26 (IBM Corp.). Statistical significance was set at *p* < 0.05.

## RESULTS

### Demographic data

No patients were lost to follow‐up during the 24–36‐month observation period. Regarding the demographic data, no significant differences were observed between the two groups in age, laterality, OA grade or additional surgical procedures (*p* = 0.061–0.855; Table 1). However, Group D had a significantly higher proportion of male patients and a greater BMI (*p* < 0.001), as well as a higher BMD (*p* = 0.003), compared with Group S.

**Table 1:** Comparison of patient demographics and baseline characteristics between Groups S and D.

Items	Group S	Group D	*p* value
Number of knees (patients)	72 (61)	82 (74)	
Age, years	60.0 [56.0−68.0]	61.0 [56.0−65.8]	0.812
Male/female sex, *n*	13/48	45/29	<0.001
Right/left knee, *n*	38/34	41/41	0.855
Height, cm	155.0 [151.8−162.6]	163.1 [156.6−170.8]	<0.001
Body weight, kg	59.0 [53.0−70.5]	71.0 [64.0–80.0]	<0.001
Body mass index, kg/m^2^	24.3 [22.4−26.7]	26.6 [24.0−28.6]	<0.001
Bone mineral density, % of YAM	85.5 [79.2−93.5]	94.5 [83.2−104.0]	0.003
Kellgren–Lawrence grade			0.061
1	2 (3%)	0 (0%)	
2	24 (33%)	15 (18%)	
3	32 (44%)	47 (57%)	
4	14 (19%)	20 (24%)	
Additional surgical treatments			
Medial meniscectomy, *n*	55 (76%)	70 (85%)	0.117
Medial meniscal repair, *n*	11 (15%)	7 (9%)	0.120
OAT, *n*	4 (5%)	6 (7%)	0.749

*Note*: Data are presented as the mean ± standard deviation for normally distributed variables or as the median [interquartile range] for non‐normally distributed variables.

Abbreviations: OAT, osteochondral autograft transfer; YAM, young adult mean.

### Radiological assessment of alignment

No significant differences were observed between the groups in preoperative radiological parameters. The intraoperative valgus correction angles were comparable between the groups (12.6° ± 2.3° in Group S vs. 11.9° ± 2.4° in Group D; *p* = 0.214). Postoperatively, no significant differences were found in any alignment parameters or patellar height (Table 2). Specifically, the postoperative HKA was 3.4° ± 3.2° in Group S and 4.3° ± 2.5° in Group D, with no significant difference.

**Table 2:** Radiological comparisons of preoperative and postoperative alignment and patellar position between Groups S and D.

Items evaluated	Group S (*n* = 72)	Group D (*n* = 82)	*p* value
Preoperative			
FTA, °	181.3 ± 2.5	182.0 ± 2.9	0.122
HKA, °	−7.4 ± 2.7	−7.7 ± 3.0	0.463
%MA, %	15.9 ± 12.4	14.1 ± 12.9	0.380
MPTA, °	83.8 ± 1.9	83.0 ± 1.9	0.070
PTS angle,	8.5 ± 2.5	8.1 ± 2.5	0.324
Insall‐Salvati ratio	0.99 ± 0.12	0.98 ± 0.10	0.325
Intraoperative			
Correction angle, °	12.6 ± 2.3	11.9 ± 2.4	0.214
Postoperative			
FTA, °	170.6 ± 3.1	169.7 ± 2.3	0.059
HKA, °	3.4 ± 3.2	4.3 ± 2.5	0.074
%MA, %	64.9 ± 12.3	64.0 ± 9.9	0.642
MPTA, °	93.5 ± 2.6	93.4 ± 2.2	0.909
PTS angle, °	6.6 ± 3.2	6.9 ± 2.4	0.480
Insall‐Salvati ratio	0.97 ± 0.10	0.97 ± 0.10	0.960

*Note*: *p* values indicate the results of comparisons between the two groups. Data are presented as the mean ± standard deviation.

Abbreviations: FTA, femorotibial angle; HKA, hip–knee–ankle angle (positive values indicate valgus alignment); %MA, percentage of the mechanical axis; MPTA, medial proximal tibial angle; PTS, posterior tibial slope.

### Progression of bone healing and time to bone union

Intra‐rater and inter‐rater reliability were 0.819 and 0.791, respectively, for bone healing type, and 0.946 and 0.953, respectively, for time to bone union.

Direct healing (Type 1), indirect healing (Type 2) and osteolysis (Type 3) were observed in 48.6%, 33.3% and 18.0% of knees in Group S and in 86.6%, 13.4% and 0.0% of knees in Group D, respectively (Table 3). The distribution of the three healing types differed significantly between the two groups (*p* < 0.001).

**Table 3:** Comparison of bone healing progression and time to union between Groups S and D.

	Group S (*n* = 72)	Group D (*n* = 82)	*p* value
Bone healing type, knees			<0.001
Type 1 (direct healing)	35 (48.6%)	71 (86.6%)	
Type 2 (indirect healing)	24 (33.3%)	11 (13.4%)	
Type 3 (osteolysis)	13 (18.0%)	0 (0.0%)	
Time to bone union,^a^ weeks	12.0 [8.0−16.0]	8.0 [8.0−12.0]	<0.001
Bone union time,^a^ knees			<0.001
≤16 weeks	53 (73.6%)	80 (97.6%)	
>16 and ≤36 weeks	15 (20.8%)	2 (2.4%)	
Non‐union	4 (5.6%)	0 (0.0%)	0.046

*Note*: Data are presented as the number of knees (percentage) for categorical variables and as the median [interquartile range] for continuous variables. *p* values indicate the results of comparisons between the two groups.

^a^ This value was analysed in the 68 knees that achieved bone union in Group S, excluding the four knees that developed non‐union.

The median time to bone union was significantly shorter in Group D (8 weeks [IQR: 8–12]) compared to Group S (12 weeks [IQR: 8–16]) (*p* < 0.001; 95% CI for the difference in medians [Group S minus Group D], 0.0–4.0 weeks). When the distribution of bone‐union timing was analysed using 16 and 36 weeks as cutoff values (Table 3), the proportion of knees that achieved union within 16 weeks was significantly higher in Group D (74% in Group S vs. 98% in Group D; *p* < 0.001).

### Complications

The incidence of delayed union was significantly lower in Group D (2.4%) than in Group S (20.8%) (risk difference: 18.4%, 95% CI: 8.6%–29.3%; *p* < 0.001) (Table 3). The odds ratio for delayed union in Group D relative to Group S was 0.095 (95% CI, 0.021–0.430). The incidence of non‐union was significantly lower in Group D (0.0%) than in Group S (5.6%) (*p* = 0.046; 95% CI for the risk difference [Group S minus Group D], 0.0%–13.4%) (Table 3). Other complications in Group D (4.9%) included one case each of transient anterior compartment syndrome, mild joint contracture, delayed wound healing and fibular non‐union. In Group S (4.2%), complications consisted of two cases of superficial infection and one case of conversion to knee arthroplasty. There was no significant inter‐group difference (*p* = 0.98; 95% CI for the risk difference, −7.2% to 8.2%).

### Clinical evaluations

The additional operative time required for SLP placement in Group D was 12.2 ± 2.1 min. Preoperatively, there were no significant differences in the JOA score, the Lysholm score or KOOS between the two groups (Table S3). At the minimum 2‐year follow‐up, all clinical scores significantly improved compared to preoperative values in both groups (*p* < 0.001; the 95% CIs for the median differences ranged from [13.2 to 22.2] to [31.3 to 46.9]). Postoperatively, the Lysholm score was slightly but significantly greater in Group D than in Group S (*p* = 0.043; 95% CI for the median difference, 0.0–5.0). However, no significant differences were observed in the JOA score or the KOOS subscales between the two groups (Table S3).

### Multivariate logistic regression analysis

The surgical technique was identified as the only independent factor significantly associated with the incidence of delayed union (aOR, 0.17; 95% CI, 0.05–0.56; *p* = 0.005). Other factors, including sex, BMI, BMD and the intraoperative correction angle, did not show significant associations with the incidence of delayed union (Table S4).

## DISCUSSION

The important findings of this study were as follows: (1) The time to bone union was substantially shorter in Group D than in Group S, and a markedly higher proportion of knees in Group D achieved bone union within 16 weeks. (2) The incidence of delayed union and non‐union was markedly lower in Group D, while the rate of other complications was comparable between the two groups. (3) Postoperative Lysholm score was slightly but significantly higher in Group D, whereas postoperative JOA and KOOS scores were comparable between the two groups. These findings demonstrated that auxiliary fixation with an SLP on the medial tibial cortex shortened the time to bone union and minimized the incidence of delayed union and non‐union compared with the conventional iVHTO procedure using single LCP fixation. In addition, this additional fixation did not increase the rates of other complications or compromise clinical outcomes of the conventional iVHTO procedure.

The findings of the present study could not be directly compared with those of earlier reports, because no previous studies have investigated the effects of auxiliary fixation on postoperative bone healing or clinical outcomes following either iVHTO or LCWHTO performed with single‐plate fixation. However, many studies have examined the effects of auxiliary fixation in plate fixation for long‐bone fractures, and they consistently report the usefulness of double‐plate fixation in promoting fracture healing [4, 6, 7, 12, 20, 32]. These reports are considered to support the validity of the present study's findings because the osteotomy site in iVHTO can be regarded as an artificially created fracture. Based on these reports, the mechanism underlying the effect of double‐plate fixation in iVHTO can be considered as follows. Double‐plate fixation, which corresponds to a fixed–fixed beam configuration in structural mechanics [9], reduces micromotion at the osteotomy site compared with single‐plate fixation, which corresponds to a cantilever beam configuration [9]. This reduction in micromotion may increase the likelihood of direct bone healing, as demonstrated in the present study, resulting in a shorter time to union and a lower incidence of delayed union and non‐union.

The clinical significance of the findings of this study warrants discussion. Delayed bone union following iVHTO or LCWHTO is a major complication, as it delays patients' return to active daily life and work, leading to social disadvantages and economic losses [26, 42]. Therefore, the present study is considered highly meaningful in demonstrating that auxiliary fixation with an SLP can minimize the occurrence of this complication after iVHTO. However, performing this minimally invasive technique required an average of 12 additional minutes of operative time, which represents one drawback of the double‐plate method. Nevertheless, this additional procedure did not increase the incidence of other complications or adversely affect clinical outcomes. Given its beneficial effect on postoperative bone healing, we believe that incorporating this additional SLP procedure has clinical value. However, to substantiate this clinical value, future studies with Level‐I evidence will be necessary. It should be noted that at the 2‑ to 3‑year postoperative evaluation, no significant intergroup differences were observed in the JOA score or KOOS, whereas the Lysholm score showed a slight but significant improvement. One possible explanation is that these scoring systems, when applied at 2–3 years postoperatively, may not adequately capture the medical, social and economic disadvantages experienced by patients during the first postoperative year due to delayed bone union. Therefore, this finding suggests that, when designing future Level‐I studies, additional clinical assessments using more appropriate scoring systems may be necessary.

A major limitation of this study is that it was conducted as a retrospective cohort study employing a sequential design. The potential drawbacks of this design include (1) temporal changes in patient characteristics over time, (2) learning curve effects and (3) differences in follow‐up duration between the two groups. Regarding the first limitation, several baseline demographic variables differed significantly between the groups in the present study. Notably, the proportion of male patients was significantly higher in Group D than in Group S. The significant intergroup differences in height, body weight, BMI and BMD are considered to reflect this difference in sex distribution. The higher proportion of male patients in Group D likely represents a temporal shift in the sex distribution of medial OA patients seeking HTO at our institution. To assess the potential influence of differences in sex and other related factors on bone healing in the present study, we performed a multivariate logistic regression analysis. Sex, BMI and BMD were not significantly associated with the incidence of delayed union. Furthermore, many previous studies have not identified sex, BMI or BMD as significant risk factors for delayed union after HTO [5, 9, 11, 17]. Therefore, we believe that the results of the present study were not influenced by these intergroup differences. Regarding the second limitation, there is a potential concern that the iVHTO procedure with lateral plate fixation performed in both groups may have been affected by learning curve effects. However, the two surgeons who operated on the patients in this study had more than 10 years of experience with the iVHTO procedure and had already performed over 200 cases using the single plating technique before the study began. Therefore, we believe that the clinical outcomes of the iVHTO procedure with lateral plate fixation in both groups during the study period were not influenced by learning curve effects. Regarding the third limitation, the difference in follow‐up duration between the two groups is a potential concern. However, in this study, for patients whose follow‐up period exceeded 36 months, the data evaluated at 24–36 months postoperatively were used for comparative analysis to ensure that the observation periods were aligned between the two groups. Therefore, there was no difference in follow‐up duration between the two groups.

There are several other limitations to this study. First, the follow‐up period was relatively short, and long‐term outcomes remain unknown. Second, although the reduction in the non‐union rate from 5.6% to 0% reached statistical significance (*p* = 0.046), the corresponding 95% CI for the risk difference (0.0%–13.4%) indicates substantial uncertainty in the magnitude of this effect. This uncertainty primarily reflects the small number of non‐union events rather than the overall sample size. Further studies with larger event counts are warranted to confirm the robustness of this finding. Third, the results of this study cannot be directly applied to medial supplemental fixation using plates that differ in shape or biomechanical function from the SLP used in this study, because variations in plate design may alter the fixation mechanics and clinical performance. Fourth, the multivariate logistic regression model did not include potential risk factors such as age, smoking status or diabetes. This was because the analysis was designed to evaluate whether the clinical parameters that differed significantly between the two groups were independent predictors of postoperative delayed union, and because the current sample size of 154 knees allowed the inclusion of only five clinically relevant covariates. To identify all independent factors associated with delayed union, studies with different designs and larger patient cohorts will be required. Fifth, the cost‐effectiveness of adding auxiliary fixation was not evaluated. Accordingly, future studies should assess the clinical utility of this auxiliary fixation technique from a health‐economic perspective. Despite these limitations, we believe that the present study provides important new insights into the field of HTO.

## CONCLUSION

Auxiliary medial fixation using a SLP enhanced bone healing and minimized the incidence of delayed union or non‐union in conventional iVHTO with single lateral LCP fixation, without increasing other complications or compromising clinical outcomes.

## AUTHOR CONTRIBUTIONS

All authors have reviewed and approved the final version of the manuscript. Takuma Kaibara performed data collection, data analysis, data interpretation and drafted the manuscript. Kazunori Yasuda and Eiji Kondo developed the initial concept, provided the data, performed data interpretation and critically appraised the manuscript. Koji Yabuuchi and Masayuki Inoue performed data analysis, data interpretation, statistical analysis and critically appraised the manuscript. Norimasa Iwasaki and Tomonori Yagi performed data analysis and critically appraised the manuscript.

## FUNDING

The authors have no funding to report.

## CONFLICT OF INTEREST STATEMENT

The authors declare no conflicts of interest.

## ETHICS STATEMENT

The study was approved by the local ethics committee (Yagi Orthopaedic Hospital, Sapporo, Japan, 2017‐04‐8), and the clinical trial was conducted in agreement with the 1964 Declaration of Helsinki and subsequent amendments. All patients signed a consent statement for the use of their data for scientific purposes.

## Supporting information

Supplementary Figure 1. Representative images of the three bone healing types. (A1–4) Type 1 (direct healing) characterized by the generation of trabecula‐like bone columns penetrating the narrow osteotomy gap; (B1–4) Type 2 (indirect healing) characterized by extraosseous bridging callus formed beyond the osteotomy site; (C1–4) Type 3 (delayed union) defined by the appearance of a distinct radiolucent zone at the osteotomy site.

Supplementary Table 1. Criteria for classification of radiological progression of bone healing at the lateral osteotomy site after iVHTO, as reported by Yabuuchi et al.[42].

Supplementary Table 2. Modified radiographic union score for Tibial fractures (RUST). On AP and lateral radiographs, bone healing was evaluated at the medial, lateral, anterior, and posterior cortices. Bone union in an osteotomy gap was assessed using CT images. The gap was divided into four zones: antero‐central, postero‐central, antero‐lateral, and postero‐lateral. The following score was assigned to each cortex or zone. The scores for the four cortices or four zones were summed (maximum 12 points) at each follow‐up.

Supplementary Table 3. Comparison of preoperative and postoperative clinical outcomes between Groups S and D. Data are presented as the median [interquartile range]. P values indicate the results of comparisons between Group S and Group D. Abbreviations: JOA, Japanese Orthopaedic Association; KOOS, Knee Injury and Osteoarthritis Outcome Score; ADL, activities of daily living; Sport/Rec, function in sport and recreation; QOL, knee‐related quality of life.

Supplementary Table 4. Multivariate logistic regression analysis performed to determine independent risk factors for postoperative delayed bone healing after inverted V‐shaped high tibial osteotomy. Abbreviations: BMI, body mass index; BMD, bone mineral density; YAM, Young Adult Mean.

## Data Availability

The data that support the findings of this study are available in the supporting information of this article.
