# Supplementary material for: Auxiliary medial small locking plate fixation enhances bone healing and minimizes delayed union in inverted V‐shaped high tibial osteotomy: A comparative study of 154 knees
Source: J Exp Orthop. 2026 Sep 23;13(3):e70919. doi: 10.1002/jeo2.70919 (PMC13598819; doi:10.1002/jeo2.70919)
Supplement: Supplementary file 1 [file JEO2-13-e70919-s001.docx]

**Supplementary Table 2.** Modified radiographic union score for Tibial fractures (RUST). On AP and lateral radiographs, bone healing was evaluated at the medial, lateral, anterior, and posterior cortices. Bone union in an osteotomy gap was assessed using CT images. The gap was divided into four zones: antero-central, postero-central, antero-lateral, and postero-lateral. The following score was assigned to each cortex or zone. The scores for the four cortices or four zones were summed (maximum 12 points) at each follow-up.

| Characteristics in observations | Score (Point) |
| --- | --- |
| Radiographic scoring to evaluate extracortical bone union  Visible osteotomy line without any callus formation  Visible osteotomy line with callus formation  Invisible osteotomy line or complete bridging callus | 1  2  3 |
| CT‑based scoring of bone union within the osteotomy gap  No trabecular formation in a gap  Sparse trabecular bridging in a gap  Abundant trabecular bridging in a gap | 1  2  3 |
