# Supplementary material for: Auxiliary medial small locking plate fixation enhances bone healing and minimizes delayed union in inverted V‐shaped high tibial osteotomy: A comparative study of 154 knees
Source: J Exp Orthop. 2026 Sep 23;13(3):e70919. doi: 10.1002/jeo2.70919 (PMC13598819; doi:10.1002/jeo2.70919)
Supplement: Supplementary file 2 [file JEO2-13-e70919-s002.docx]

**Supplementary Table 3.** Comparison of preoperative and postoperative clinical outcomes between Groups S and D. Data are presented as the median [interquartile range]. P values indicate the results of comparisons between Group S and Group D. Abbreviations: JOA, Japanese Orthopaedic Association; KOOS, Knee Injury and Osteoarthritis Outcome Score; ADL, activities of daily living; Sport/Rec, function in sport and recreation; QOL, knee-related quality of life.

| Items evaluated | Group S (n = 72) | Group D (n =82) | *P* value |
| --- | --- | --- | --- |
| Preoperative |  |  |  |
| JOA score (points) | 70.0 [65.0 - 75.0] | 67.0 [55.0 - 75.0] | 0.272 |
| Lysholm score (points) | 64.0 [53.0 - 71.5] | 60.0 [48.0 - 69.0] | 0.142 |
| KOOS |  |  |  |
| Pain | 54.2 [41.7 - 66.8] | 55.6 [38.9 - 69.4] | 0.981 |
| Symptoms | 60.7 [38.4 - 75.0] | 60.7 [42.9 - 71.4] | 0.798 |
| ADL | 72.8 [58.1 - 82.4] | 72.0 [61.8 - 83.8] | 0.802 |
| Sport/Rec | 32.5 [15.0 - 50.0] | 30.0 [15.0 - 50.0] | 0.951 |
| QOL | 31.2 [25.0 - 45.3] | 31.2 [18.8 - 43.8] | 0.582 |
| Postoperative |  |  |  |
| JOA score (points) | 90.0 [90.0 - 95.0] | 95.0 [90.0 - 95.0] | 0.086 |
| Lysholm score (points) | 94.0 [89.5 - 96.5] | 95.0 [90.0 - 99.0] | 0.043 |
| KOOS |  |  |  |
| Pain | 91.7 [80.6 - 94.4] | 91.7 [83.3 - 94.4] | 0.887 |
| Symptoms | 86.0 [75.0 - 92.9] | 82.1 [75.0 - 92.9] | 0.287 |
| ADL | 91.2 [85.3 - 95.6] | 92.6 [84.9 - 98.5] | 0.648 |
| Sport/Rec | 65.0 [45.0 - 85.0] | 67.5 [53.8 - 76.2] | 0.970 |
| QOL | 75.0 [62.5 - 81.2] | 68.8 [62.5 - 87.5] | 0.912 |
