# Supplementary material for: Auxiliary medial small locking plate fixation enhances bone healing and minimizes delayed union in inverted V‐shaped high tibial osteotomy: A comparative study of 154 knees
Source: J Exp Orthop. 2026 Sep 23;13(3):e70919. doi: 10.1002/jeo2.70919 (PMC13598819; doi:10.1002/jeo2.70919)
Supplement: Supplementary file 3 [file JEO2-13-e70919-s003.docx]

**Supplementary Table 4.** Multivariate logistic regression analysis performed to determine independent risk factors for postoperative delayed bone healing after inverted V-shaped high tibial osteotomy. Abbreviations: BMI, body mass index; BMD, bone mineral density; YAM, Young Adult Mean.

| Variable | Adjusted Odds Ratio | 95% Confidence Interval | *P* value |
| --- | --- | --- | --- |
| Surgical technique (Single vs. Double) | 0.17 | 0.05 – 0.56 | 0.005 |
| Male sex | 1.81 | 0.54 – 6.45 | 0.343 |
| BMI | 1.12 | 0.98 – 1.30 | 0.111 |
| BMD (% of YAM) | 1.01 | 0.97 – 1.05 | 0.600 |
| Correction angle | 1.02 | 0.80 – 1.30 | 0.859 |
