# Supplementary material for: Auxiliary medial small locking plate fixation enhances bone healing and minimizes delayed union in inverted V‐shaped high tibial osteotomy: A comparative study of 154 knees
Source: J Exp Orthop. 2026 Sep 23;13(3):e70919. doi: 10.1002/jeo2.70919 (PMC13598819; doi:10.1002/jeo2.70919)
Supplement: Supplementary file 4 [file JEO2-13-e70919-s005.docx]

**Supplementary Table 1.** Criteria for classification of radiological progression of bone healing at the lateral osteotomy site after iVHTO, as reported by Yabuuchi et al.[42].

| Type | Characteristics of radiographic findings | Characteristics of CT images |
| --- | --- | --- |
| 1 | - The osteotomy line became indistinct at 5 weeks and invisible at 8 weeks.  - Extraosseous bridging callus was rarely formed. | - Trabecula-like bone columns penetrating the narrow osteotomy gap were found at 5 weeks and increased at 8–12 weeks.  - Extraosseous bridging callus was rarely formed. |
| 2 | - The osteotomy line became indistinct at 8 weeks and invisible at 12 weeks.  - Extraosseous bridging callus was visible at 5 weeks and abundantly formed at 8–12 weeks. | - No trabecula-like bone columns were generated in the gap.  - Extraosseous bridging callus was visible at 5 weeks and abundantly formed at 8–12 weeks. |
| 3 | - The osteotomy line remained distinct at 5 weeks and became radiolucent at 8 weeks.  - Extraosseous bridging callus formation was visible at 8 weeks and abundantly formed at 12–24 weeks. | - An osteolytic zone was observed in the tibia at 5 weeks and progressively enlarged at 8–12 weeks.  - Extraosseous bridging callus was visible at 5 weeks and abundantly formed at 8–24 weeks. |
